# Simultaneous detection of pathogens and antimicrobial resistance genes with the open source, cloud-based, CZ ID platform

**DOI:** 10.1186/s13073-025-01480-2

**Published:** 2025-05-06

**Authors:** Dan Lu, Katrina L. Kalantar, Abigail L. Glascock, Victoria T. Chu, Estella S. Guerrero, Nina Bernick, Xochitl Butcher, Kirsty Ewing, Elizabeth Fahsbender, Olivia Holmes, Erin Hoops, Ann E. Jones, Ryan Lim, Suzette McCanny, Lucia Reynoso, Karyna Rosario, Jennifer Tang, Omar Valenzuela, Peter M. Mourani, Amy J. Pickering, Amogelang R. Raphenya, Brian P. Alcock, Andrew G. McArthur, Charles R. Langelier

**Affiliations:** 1 Chan Zuckerberg Initiative, Redwood City, CA USA; 2 Chan Zuckerberg Biohub, San Francisco, CA USA; 3 Division of Infectious Diseases, University of California, San Francisco, San Francisco, CA USA; 4 Nova Southeastern University, Fort Lauderdale, FL USA; 5 Department of Pediatrics, University of Arkansas for Medical Sciences, Little Rock, AR USA; 6 Arkansas Children’s, Little Rock, AR USA; 7 University of California, Berkeley, Berkeley, CA USA; 8 Department of Biochemistry & Biomedical Sciences, McMaster University, Hamilton, ON Canada; 9 Michael G. DeGroote Institute for Infectious Disease Research, McMaster University, Hamilton, ON Canada

**Keywords:** Antimicrobial resistance, Metagenomics, Whole-genome sequencing, Chan Zuckerberg ID, CZ ID

## Abstract

**Background:**

Antimicrobial resistant (AMR) pathogens represent urgent threats to human health, and their surveillance is of paramount importance. Metagenomic next-generation sequencing (mNGS) has revolutionized such efforts, but remains challenging due to the lack of open-access bioinformatics tools capable of simultaneously analyzing both microbial and AMR gene sequences.

**Results:**

To address this need, we developed the Chan Zuckerberg ID (CZ ID) AMR module, an open-access, cloud-based workflow designed to integrate detection of both microbes and AMR genes in mNGS and single-isolate whole-genome sequencing (WGS) data. It leverages the Comprehensive Antibiotic Resistance Database and associated Resistance Gene Identifier software, and works synergistically with the CZ ID short-read mNGS module to enable broad detection of both microbes and AMR genes from Illumina data. We highlight diverse applications of the AMR module through analysis of both publicly available and newly generated mNGS and single-isolate WGS data from four clinical cohort studies and an environmental surveillance project. Through genomic investigations of bacterial sepsis and pneumonia cases, hospital outbreaks, and wastewater surveillance data, we gain a deeper understanding of infectious agents and their resistomes, highlighting the value of integrating microbial identification and AMR profiling for both research and public health. We leverage additional functionalities of the CZ ID mNGS platform to couple resistome profiling with the assessment of phylogenetic relationships between nosocomial pathogens, and further demonstrate the potential to capture the longitudinal dynamics of pathogen and AMR genes in hospital acquired bacterial infections.

**Conclusions:**

In sum, the new AMR module advances the capabilities of the open-access CZ ID microbial bioinformatics platform by integrating pathogen detection and AMR profiling from mNGS and single-isolate WGS data. Its development represents an important step toward democratizing pathogen genomic analysis and supporting collaborative efforts to combat the growing threat of AMR.

**Supplementary Information:**

The online version contains supplementary material available at 10.1186/s13073-025-01480-2.

## Background

Antimicrobial resistance (AMR) is responsible for an estimated 1.27 million global deaths annually [1], and is on track to cause 10 million deaths a year by 2050, becoming a leading cause of global mortality [2]. Furthermore, the World Health Organization has declared AMR to be one of the top ten global public health threats facing humanity [3].

A critical step in combating AMR is the development and implementation of new methods and analysis tools for genomic detection and surveillance of AMR microbes with high resolution and throughput. Whole-genome sequencing (WGS) of cultured bacterial isolates and direct metagenomic next-generation sequencing (mNGS) of biological and environmental samples have emerged at the forefront of technological advances for AMR surveillance [4, 5]. Several tools and databases have been developed over the past decade to enable the detection of AMR genes from both single-isolate WGS and mNGS data. These include ResFinder [6], ResFinderFG [7], the Comprehensive Antibiotic Resistance Database (CARD) [8, 9], ARG-ANNOT [10], SRST2 [11], AMRFinderPlus and the Reference Gene Catalog by NCBI [12], among others.

Effective surveillance for resistant pathogens requires not only detecting AMR genes, but also detecting their associated microbes. Despite this, each task has traditionally been approached separately in bioinformatics pipelines, with few available tools enabling simultaneous evaluation of both. The Chan Zuckerberg ID (CZ ID) mNGS module, for instance, was developed in 2017 to democratize access to metagenomic data analysis through a free, no-code, cloud-based workflow, but has had limited AMR assessment capabilities [13].

Realizing the unmet need for, and potential impact of, a single bioinformatics tool integrating the detection of both AMR genes and microbes, we sought to add AMR analysis capabilities to the open-access CZ ID mNGS pipeline. Here, we report the development of a new AMR module within the CZ ID web platform, which leverages CARD’s Resistance Gene Identifier (RGI) algorithm and comprehensive database [14] to support openly accessible AMR detection and analysis. We demonstrate its utility across both single-isolate WGS and mNGS data, and in clinical and environmental samples, and demonstrate the value of enriching AMR findings through simultaneous unbiased profiling of microbes.

## Implementation

### AMR gene and variant detection using the CZ ID AMR module

The AMR module is incorporated into the CZ ID platform (https://czid.org) [13] and allows researchers to upload FASTQ files from both mNGS and single-isolate WGS Illumina data. Once uploaded, the module automatically processes samples in the cloud using Amazon Web Services (AWS) infrastructure, eliminating the need for users to download and install software or maintain high-performance computing resources. A sample with 50 million reads typically takes less than 5 h to process after upload. The web-based platform makes analysis of AMR datasets accessible even to researchers with limited bioinformatics or computational expertise, and an extensive Help Center [15] contains articles to support every aspect of the analysis such as pipeline set up, filtering host reads, sample quality control, and result interpretation. The data are securely stored and never shared with other CZ ID users unless the users choose to make the project public or share with collaborators. The complete privacy policy can be found at https://czid.org/privacy.

Underlying the AMR module is CARD, a comprehensive, continually curated database of AMR genes and their variants, linked to gene family, resistance mechanism, and drug class information [8, 9]. CARD supports various AMR models such as the *protein homolog model* which detects AMR genes whose presence and expression alone is sufficient to confer resistance and the *protein variant model* which identifies specific mutations that confer resistance in over 40 species. The AMR module specifically leverages the CARD Resistance Gene Identifier (RGI) tool [14] to match short reads or contigs to AMR gene reference sequences in the CARD database, returning metrics such as gene coverage and percent identity.

RGI was recently benchmarked against nine other commonly used AMR detection tools and performed well across a diversity of metrics [16]. The precision (0.988–0.993) and accuracy (0.982–0.983) of RGI also ranked among the best, while the specificity (0.079–0.200) was on the lower end of the spectrum [16]. The high sensitivity of RGI enables detection of AMR genes even in sparse datasets, common with metagenomics. Importantly, while the specificity of RGI was low, the tool does provide several metrics that can be used to filter the dataset and improve specificity by retaining only the highest confidence hits.

CARD also maintains a Resistomes, Variants, & Prevalence database of predicted AMR alleles and their distribution among pathogens and plasmids [17, 18]. This database provides information linking AMR genes to specific species, and can be used for k-mer-based pathogen-of-origin prediction, a beta feature implemented in RGI [14]. This feature leverages k-mers that are uniquely found within AMR alleles of individual pathogen species, pathogen genera, pathogen-restricted plasmids, or promiscuous plasmids. These k-mers are then used to predict pathogen-of-origin and a chromosomal versus plasmid AMR gene location based on input AMR sequences from short reads or contigs.

**Fig. 1:**
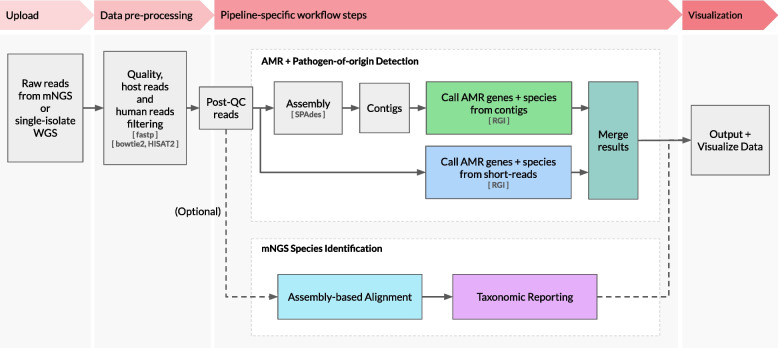
High-level flow diagram highlighting the integrated AMR and mNGS modules within the CZ ID platform. A more detailed diagram is provided in Additional file 1: Fig. S1

The CZ ID AMR module automates the running of a containerized WDL workflow that strings together multiple steps and informatics tools to enable efficient data processing and accurate resistome profiling (Fig. 1, Additional file 1: Fig. S1). The workflow shares the same preprocessing steps as the existing CZ ID mNGS module. Briefly, it accepts raw FASTQ files from Illumina mNGS (from DNA or RNA) or single-isolate WGS samples and will process up to 75 million single-end or 150 million paired-end reads per sample. Low quality and low complexity reads are first removed with fastp [19]. Host reads are removed with Bowtie2 [20] followed by HISAT2 [21] alignments against reference genomes. Regardless of host, human sequences are also removed using Bowtie2 and HISAT2 alignments against the human genome. Duplicate reads are then filtered out using CZID-dedup [22].

The resulting quality- and host-filtered reads are subsampled to 1 million single-end reads or 2 million paired-end reads to limit the resources required for compute-intensive downstream alignment steps. In the AMR workflow, to accommodate targeted mNGS protocols designed to amplify many copies of low abundance AMR genes, duplicate reads are then added back prior to further processing. To support reproducible analysis, each project on CZ ID is associated with the specific pipeline and database version used by the first sample uploaded to the project, with the version information provided on both the upload and sample details pages.

There are two parallel approaches for AMR gene detection (Fig. 1, Additional file 1: Fig. S1). In the “contig” approach, the short reads are assembled into contiguous sequences (contigs) using SPAdes [23], and the contigs are subsequently sent to RGI (with command “rgi main -a BLAST”) for AMR gene detection based on sequence similarity and mutation mapping. In the “read” approach, the short reads are directly sent to RGI (with command “rgi bwt -a kma”) for read mapping by KMA [24] to CARD reference sequences. In both approaches, the assembled contigs or reads containing AMR genes are also sent to RGI (with command “rgi kmer_query”) for pathogen-of-origin detection.

### AMR module result output

**Fig. 2:**
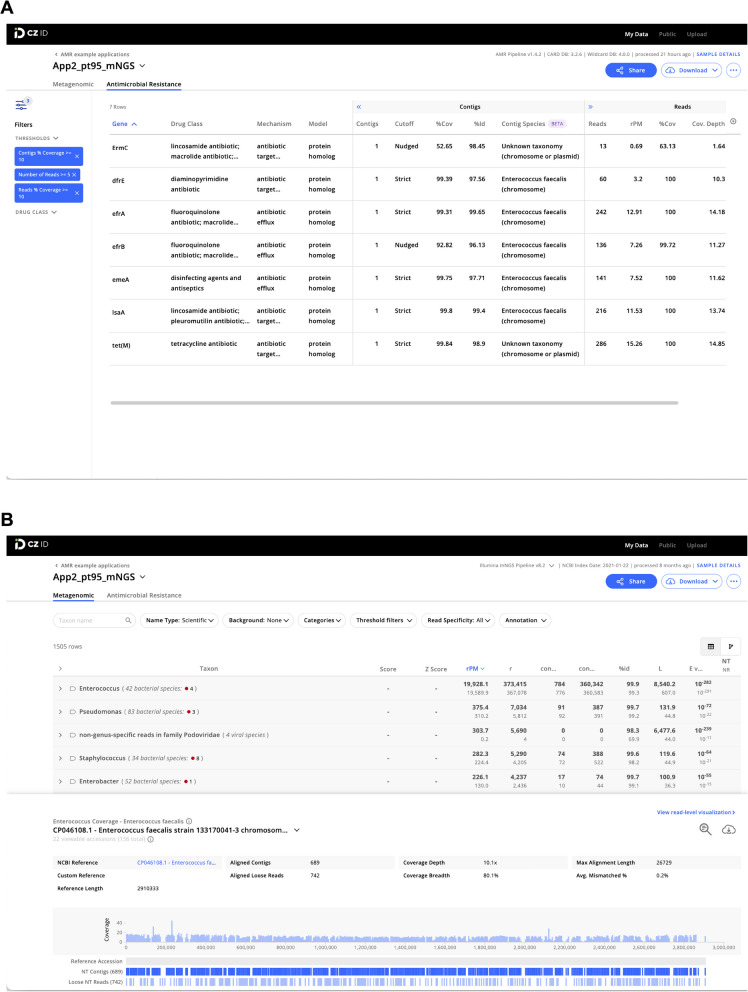
Examples of CZ ID web tool sample reports. **A** The report in the AMR module with a filter of Number of Reads ≥ 5 and Reads/Contig percent coverage ≥ 10% applied to the detected AMR genes. **B** The report in the mNGS module showing the list of detected species (top) and the coverage visualization for one species (bottom). Details about report metrics are discussed in the main text and CZ ID help center https://help.czid.org/

The AMR module displays results in an interactive table, facilitating viewing, sorting, and filtering. The table is organized in three collapsible vertical sections: (1) general information, (2) Contigs, and (3) Reads (Fig. 2A). The general information section includes columns “Gene” (showing the Antibiotic Resistance Ontology (ARO) term CARD assigned for each AMR gene and its protein product) and “Gene Family,” as well as information on the antibiotic(s) against which the gene confers resistance (“Drug Class” and “High-level Drug Class”), resistance mechanism (“Mechanism”), and model used to identify resistance (“Model”). Clicking on the AMR gene name will reveal a description and web hyperlinks to CARD, NCBI, and PubMed entries.

The “Contigs” section includes the number of contigs that map to each AMR gene (“Contigs”), cutoff based on BLAST bit-score (“Cutoff”), percentage of the AMR gene covered by all contigs (“%Cov”), percent identity of the covered region (“%Id”), and pathogen-of-origin prediction based on contigs (“Contig Species”). The “Reads” section includes metrics corresponding to the number of reads mapping to the AMR gene (“Reads”), relative abundance of the AMR gene in reads per million reads sequenced (“rpM”), percentage of AMR gene covered by sequencing reads (“%Cov”), average depth of reads aligned across the gene (“Cov. Depth”), average depth of reads aligned across the gene per million reads sequenced (“dpM”), and a pathogen-of-origin prediction based on reads (“Read Species”). All columns can be sorted, and numerical metrics can be further filtered using user specified thresholds.

Results files at each stage of the pipeline can be downloaded for inspection or additional downstream analysis. These files include quality- and host-filtered reads, assembled contigs, AMR annotations and corresponding metrics in tabular format, and all output files from CARD RGI. The contigs as well as short reads mapped to each individual AMR gene can also be downloaded. The AMR module does not provide heatmap plotting functionality at the moment, but users can download the results and use CZ ID’s public scripts to generate heatmaps [25].

### Quality filtering for AMR gene predictions

One challenge with mNGS-based AMR surveillance is interpretation of results. Aside from metrics to support standard sample quality control and remove low quality samples [13], the CZ ID AMR module provides key quantitative metrics including rpM, percent coverage of the AMR gene, and dpM to enable assessments of relative abundance and the confidence of AMR gene assignments. Additionally, for AMR detection using contigs, the “Cutoff” column which reports RGI’s stringency thresholds based on CARD’s curated bit-score cut-offs can provide valuable insight into AMR gene alignment confidence. Here, “Perfect” indicates perfect or identical matches to the curated reference sequences and mutations in CARD while “Strict” indicates matches to variants of known AMR genes, including a secondary screen for key mutations. Finally, the terminology “Nudged” is adopted by the CZ ID module to indicate more distant homologs (matched via RGI’s “Loose” paradigm) with at least 95% identity to known AMR genes, which is ideal for discovery but is more likely to return false-positive hits. Given that a consensus approach has yet to be developed for quantifying and interpreting AMR genes from mNGS and single-isolate WGS data, the CZ ID AMR module provides comprehensive information that can be subsequently filtered or otherwise optimized based on the goals of a given analysis.

### Microbial profiling using the CZ ID mNGS module

The CZ ID mNGS module, which has undergone several updates since first described [13], preprocesses the uploaded reads and then proceeds to assembly-based alignment to produce taxonomic relative abundance profiles for each sample (Fig. 1, Additional file 1: Fig. S1). Briefly, the non-host reads output by the quality- and host-filtering steps (as described above) are aligned to the NCBI nucleotide (NT) and protein (NR) databases using minimap2 [26] and DIAMOND [27], respectively, to identify putative short-read alignments. Then, reads are assembled into contigs using SPAdes [23] and contigs are re-aligned to the set of putative accessions using BLAST [28] to improve specificity. Finally, alignments are used to identify taxa of origin, which are tallied into relative abundance estimates [13]. The web interface provides various reports with metrics including reads per million (“rpM”), number of reads (“r”), number of contigs (“contig”), number of reads in the contigs (“contig r”), percent identity (“%id”), and average length of alignment (“L”), alongside visualizations and download options to support the analysis and exploration of results (Fig. 2B).

### Connecting pathogens and AMR genes

The CZ ID platform enables simultaneous data analysis of microbe and AMR genes from a single data upload via the mNGS and AMR modules. This provides complementary, but distinct, microbial and AMR gene profiles from a given sample or dataset. The mNGS module does not provide any direct link between species calls and AMR genes from the AMR module, although in cases where a single bacterial pathogen comprises the majority of reads in a metagenomic sample, this may be inferred.

Conversely, the AMR module provides two ways to help connect AMR genes to their potential source microbes. Note that neither approach is definitive, and each should be considered collectively with other available evidence. First, each AMR gene returned in the report table is hyperlinked to its corresponding CARD webpage, where the Resistomes section reports all species in which the gene and its variants have been identified by RGI. This is based on CARD Resistomes, Variants, & Prevalence data that was generated by searching for AMR sequences in genomes, plasmid, or genomic islands for more than 400 pathogens of interest using RGI [17, 18]. Secondly, the AMR module returns results from a pathogen-of-origin analysis conducted by RGI’s beta feature in the “Contig/Read Species” columns [14], which maps k-mers derived from reads or contigs containing the AMR gene of interest against AMR alleles in the Resistomes, Variants, & Prevalence database. This second approach is particularly useful for identifying the source species in cases when the first CARD Resistomes section lists multiple species or genera by potentially capturing the differences among alleles. The pathogen-of-origin tool also attempts to predict whether an AMR gene is found on a chromosome or plasmid, based on CARD annotations. Because many AMR genes reside on plasmids that can be exchanged between species, species-of-origin predictions for AMR genes found on plasmids should be interpreted with caution. It is worth noting that in the pathogen-of-origin analysis, only AMR gene sequences are used for species prediction, as opposed to species identification using complete reference genome sequences in the mNGS module.

### Sharing results for collaboration

Projects on CZ ID can be shared with specific users or made public to all users. Everyone with access to the project can view or download the results and perform data filtering or other analyses. All data and results for this paper can be accessed by searching for a project named “AMR example applications” among public projects at https://czid.org.

### Comparative utility of the CZ ID AMR module

A feature comparison between the CZ ID AMR module and other commonly used AMR identification tools [12, 29–34] demonstrates the strengths of the CZ ID AMR module (Table 1). Notably, the CZ ID AMR module supports a seamlessly integrated workflow from raw sequencing reads to QC, host filtering, contig assembly, and AMR detection in both reads and contigs. Uniquely, it allows for side-by-side comparison of results from reads and contigs.

**Table 1:**
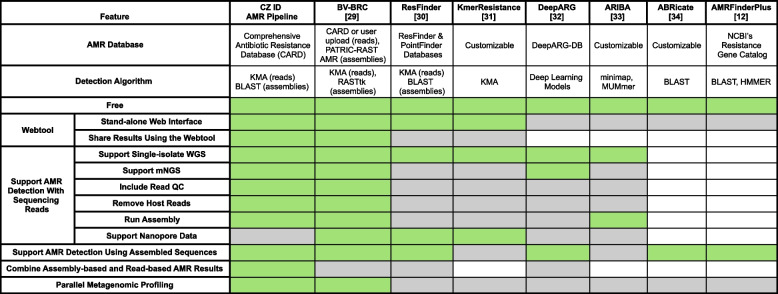
Feature comparison of AMR identification tools. Cell colors correspond to the presence of a given feature in each tool: green—present, gray—absent, white—not applicable

### Samples for example applications

We demonstrate the utility of CZ ID AMR module with 5 example applications. Application 1 used bacterial isolate WGS and plasma mNGS data from two patients with transfusion-related sepsis [35]. Application 2 used bacterial isolate WGS data of surveillance skin swabs collected from 40 babies in a neonatal intensive care unit (data unpublished). Application 3 used RNA-seq data from two critically ill patients with acute infections [36, 37]. Application 4 used time course RNA-seq data from a critically ill patients with two tandem infections [38, 39]. Application 5 used mNGS data from a wastewater surveillance study [40]. We obtained raw FASTQ files from previous studies, either from the authors or public repositories, and uploaded them to the CZ ID pipeline to be processed through both the AMR and mNGS modules. For previously unpublished data used in Application 2 and Application 4, host-depleted FASTQ files were submitted to NCBI Sequence Read Archive (SRA) with BioProject accession PRJNA1086943. More details for sample and data processing can be found in Additional file 1.

## Results

### Application 1: identification of AMR genes from single-isolate WGS and mNGS data

**Fig. 3:**
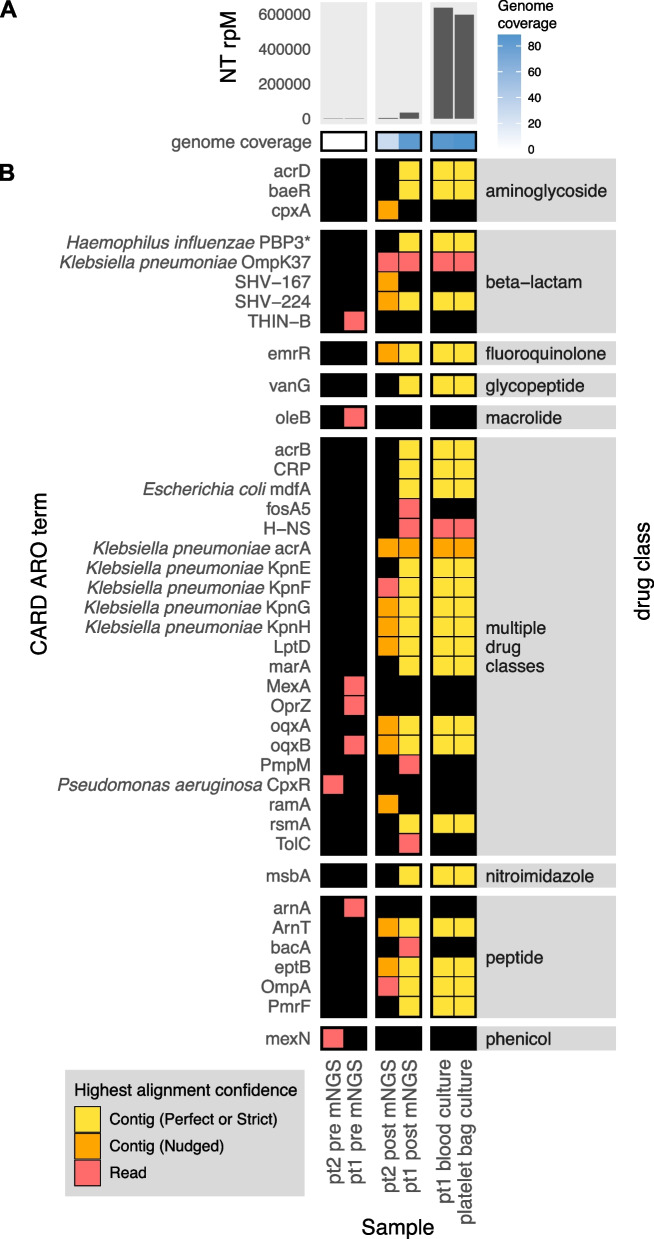
Combining pathogen detection and AMR gene profiling of mNGS and single-isolate WGS data to investigate *Klebsiella pneumoniae* transfusion-related sepsis. **A** Abundance and genome coverage of *Klebsiella pneumoniae* from direct mNGS of plasma or serum samples versus WGS of cultured bacterial isolates. **B** AMR genes detected in each sample. * denotes AMR gene(s) for which resistance originates due to point mutations (as opposed to presence/absence of the gene); these were detected by the “protein variant model” in CARD and the gene name shown is a representative reference gene containing the mutations known to lead to resistance. Legend: NT rPM = reads mapping to pathogen in the NCBI NT database per million reads sequenced. Contig = contiguous sequence. Strict/Perfect/Nudged refers to RGI’s alignment stringency threshold. If one gene was detected through multiple contigs or reads, the highest alignment confidence among them is shown on the plot (see methods in Additional file 1). “pt1” = patient 1, “pt2” = patient 2. “pre” = pre-transfusion, “post” = post-transfusion

To demonstrate the CZ ID AMR module’s utility for detecting bacterial pathogens and their AMR genes in both single-isolate WGS and mNGS data, we leveraged data from a recent investigation of transfusion-related sepsis [35]. In this study, two immunocompromised patients received platelet units originating from a single donor. Both developed septic shock within hours after the transfusion, with blood cultures from patient 1, who did not survive, returning positive for *Klebsiella pneumoniae*. Patient 2, who was receiving prophylactic antibiotic therapy at the time of the transfusion, survived, but had negative blood cultures. Direct mNGS of post-transfusion blood samples from both patients revealed a large increase in reads mapping to *Klebsiella pneumoniae*, a pathogen which was later also identified from culture of residual material from the transfused platelet bag (Fig. 3A) [35]. While blood mNGS data yielded less coverage of the *K. pneumoniae* genome compared to WGS of the cultured isolates, mNGS of patient 1’s post-transfusion plasma sample recovered all the AMR genes found by WGS of cultured isolates (Fig. 3B). Even in patient 2, whose blood sample had fewer reads mapping to *K. pneumoniae*, most AMR genes found in the cultured isolates were still able to be identified using the RGI “Nudged” threshold.

### Application 2: comprehensive profiling of pathogens and AMR genes in the setting of a hospital outbreak

**Fig. 4:**
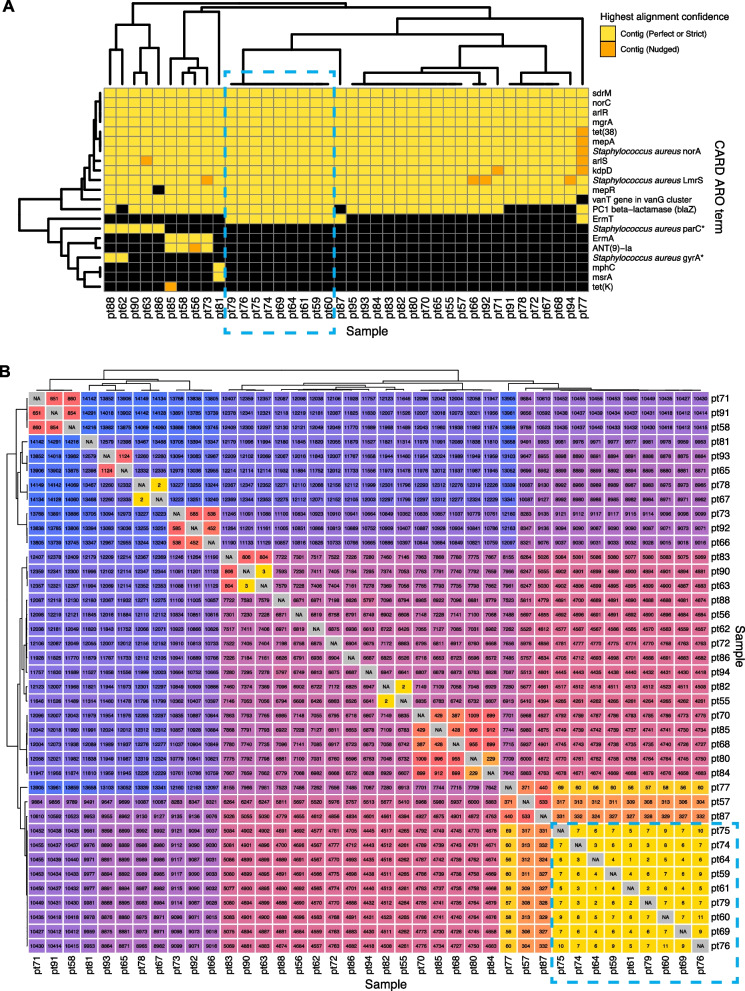
Outbreak investigation pairing WGS of methicillin susceptible *Staphylococcus aureus* isolates and mNGS of surveillance skin swabs from babies in a neonatal intensive care unit. **A** Unsupervised clustering of AMR gene profiles from single-isolate WGS data reveals a cluster of related isolates indicated by the dashed-line box. **B** Matrix of single nucleotide polymorphism (SNP) distances between each sequenced isolate confirms the genetic relatedness of this cluster, which is highlighted by a dashed-line box

To demonstrate how the CZ ID AMR module can facilitate deeper insights into pathogen and AMR transmission in hospitals, we evaluated single-isolate WGS and mNGS data from surveillance skin swabs collected from 40 babies in a neonatal intensive care unit (NICU). The swabs were collected to evaluate for suspected transmission of methicillin-susceptible *Staphylococcus aureus* (MSSA) between patients. WGS of the MSSA isolates followed by implementation of the AMR module demonstrated many shared AMR genes and revealed a cluster of nine samples with identical AMR profiles (Fig. 4A). Subsequent phylogenetic assessment using split k-mer analysis with SKA2 [41] revealed that samples within this cluster differed by less than 11 single nucleotide polymorphisms (SNP) across their genomes, consistent with an outbreak involving *S. aureus* transmission between patients (Fig. 4B).

**Fig. 5:**
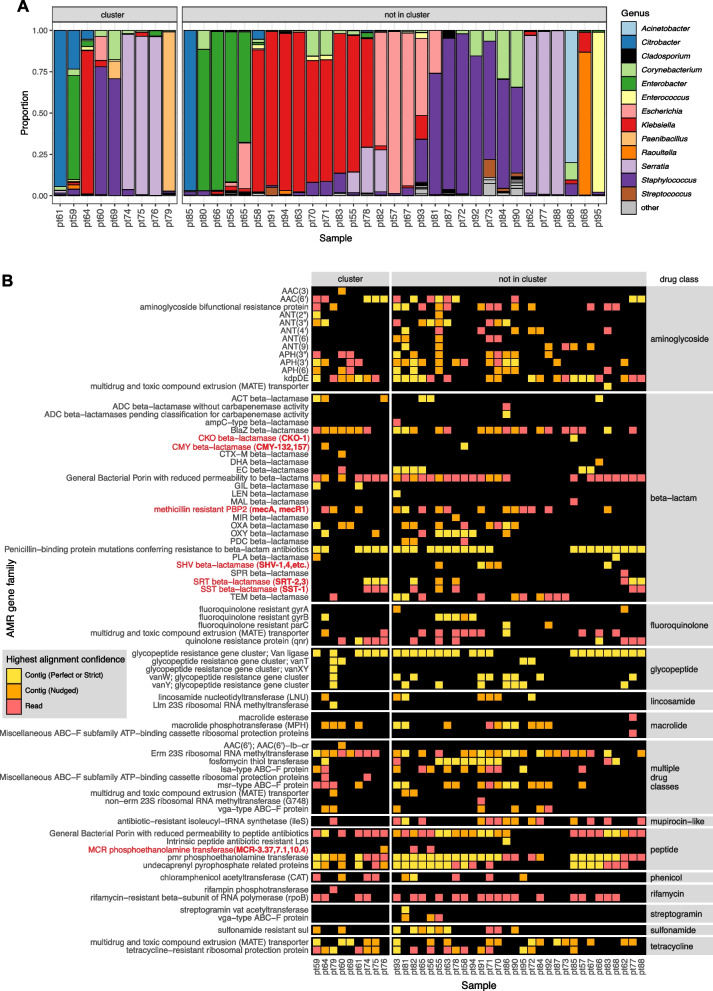
Bacterial genera and AMR gene families detected by mNGS of skin swabs from babies in a neonatal intensive care unit. **A** mNGS of swab samples demonstrated a diversity of genera in both samples from patients within an outbreak cluster of genetically related *S. aureus*, as well as in those from patients outside of the cluster. **B** mNGS analysis revealed a greater number and type of AMR gene families versus those identified by WGS of *S. aureus* isolated in culture from the swabs in Fig. 4A. Selected AMR gene families of high public health concern are highlighted in red with the specific genes (shown using CARD ARO terms) detected in parenthesis

Within this cluster of patients, we considered whether other bacterial species in the microbiome were also being exchanged in addition to the *S. aureus*. Intriguingly, mNGS analysis of the direct swab samples from which the *S. aureus* isolates were selectively cultured revealed a diversity of bacterial taxa, many of which were more abundant than *S. aureus*. These included several healthcare-associated obligate and contextual pathogens that were never identified using the selective culture-based approach, such as *Enterobacter*, *Citrobacter*, *Klebsiella*, and *Enterococcus* species. mNGS also demonstrated that each sample had a distinct microbial community composition even among samples from the cluster, indicating that only *S. aureus* and potentially a subset of other species were actually exchanged between babies, rather than the entire skin microbiome (Fig. 5A).

Further analysis of mNGS data using the AMR module also revealed a diversity of AMR genes conferring resistance to several drug classes and commonly associated with nosocomial pathogens. These included genes encoding AmpC-type inducible beta-lactamases (e.g., CKO, CMY, SST), extended spectrum beta-lactamases (e.g., SHV), and the recently emerged *mcr* genes, which confer plasmid-transmissible colistin resistance [42].

The AMR gene profiles varied greatly across the samples, both within the cluster and outside of the cluster, consistent with the observed taxonomic diversity (Fig. 5B). Together, these results revealed both inter-patient MSSA transmission in the NICU and the acquisition of AMR genes associated with nosocomial pathogens within the first months of life.

### Application 3: correlating pathogen identification with AMR gene detection

**Fig. 6:**
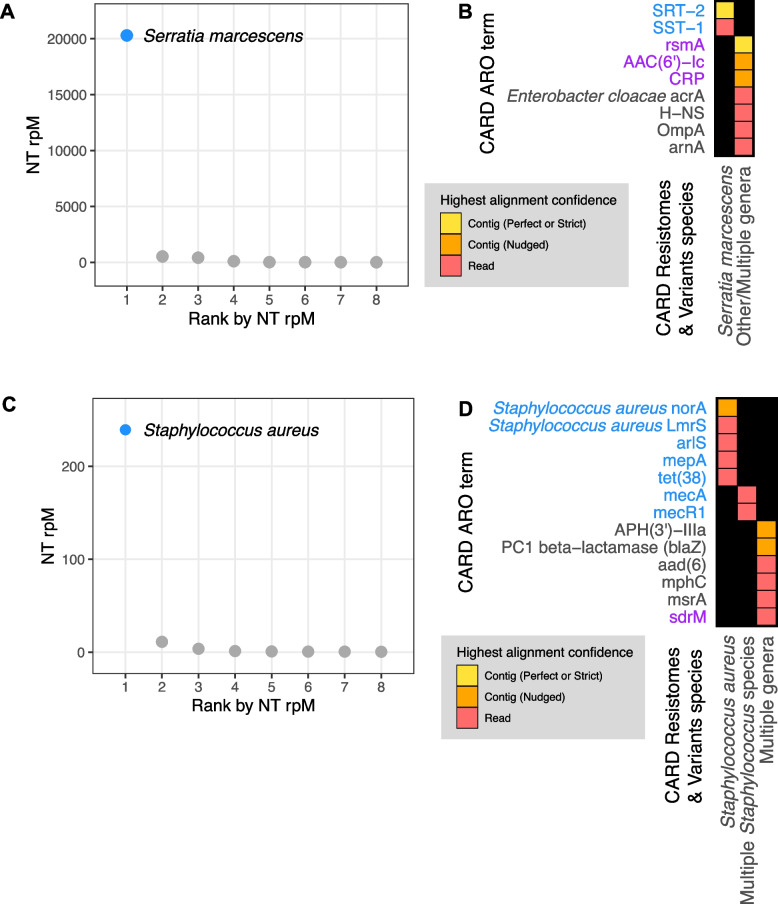
Co-detection of microbes and AMR genes in patients with critical bacterial infections. **A** Relative abundance (reads per million, rpM) of the eight most abundant taxa in the lower respiratory tract detected by RNA mNGS of tracheal aspirate from a patient with *Serratia marcescens* pneumonia. The dominant microbe is highlighted in blue. **B** AMR genes and their species prediction by the AMR module. Columns indicate the species these AMR genes and their variants are found in according to CARD Resistomes, Variants, & Prevalence database, and those found in the dominant species as in **A** are colored in blue. AMR genes that are further associated with the dominant species by the pathogen-of-origin analysis are colored in purple. **C** Relative abundance (rpM) of the eight most abundant taxa detected by plasma DNA mNGS in a patient with sepsis due to MRSA bloodstream infection. The dominant microbe is highlighted in blue. **D** AMR genes and their species prediction by the AMR module. Columns indicate the species these AMR genes and their variants are found in according to CARD Resistomes, Variants, & Prevalence database, and those found in the dominant species as in **C** are colored in blue. AMR genes that are further associated with the dominant species by the pathogen-of-origin analysis are colored in purple

Next, we aimed to integrate results from the CZ ID mNGS and AMR modules by analyzing mNGS data from critically ill patients with bacterial infections. In patient 350, who was hospitalized for *Serratia marcescens* pneumonia, RNA sequencing (RNA-seq) of a lower respiratory tract sample identified *Serratia marcescens* as the single most dominant species within the lung microbiome (Fig. 6A) [36]. Among the detected AMR sequences, based on the Resistomes, Variants, & Prevalence information from CARD, SRT-2 and SST-1 are found exclusively in *Serratia marcescens* (Fig. 6B in blue). Further analysis by the pathogen-of-origin feature in the AMR module matched the k-mers from reads and contigs containing *rsmA*, *aac(6’)-Ic*, and *crp* to *Serratia marcescens* (Fig. 6B in purple).

In patient 11,827, who was hospitalized for sepsis due to a methicillin-resistant *Staphylococcus aureus* (MRSA) blood stream infection, analysis of plasma mNGS data demonstrated that *Staphylococcus aureus* was the dominant species present in the blood sample (Fig. 6C) [37]. Among the detected AMR genes, based on Resistome & Variants information from CARD, *Staphylococcus aureus norA*, *Staphylococcus aureus lmrS*, *arlS*, *mepA*, *tet38*, *mecR1*, and *mecA* are found exclusively in staph species (Fig. 6D in blue). Pathogen-of-origin analysis further matched k-mers from the reads containing *sdrM* to *S. aureus* (Fig. 6D in purple).

### Application 4: profiling the longitudinal dynamics of pathogens and AMR genes

**Fig. 7:**
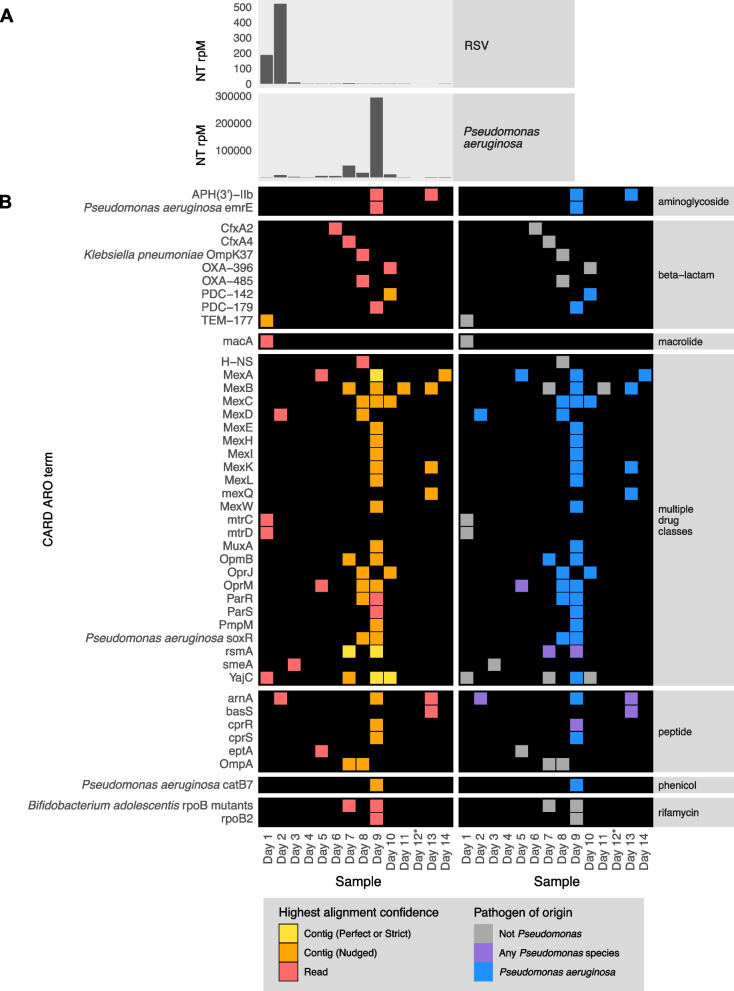
Longitudinal profiling of pathogen and AMR gene abundance in a patient hospitalized for severe respiratory syncytial virus (RSV) infection who developed *Pseudomonas aeruginosa* ventilator-associated pneumonia (VAP). **A** Relative abundance in reads per million (rpM) of RSV and *P. aeruginosa*. **B** AMR genes detected in the lower respiratory tract microbiome at each time point. Perfect or strict AMR alignments from contigs are highlighted in yellow, while those nudged are orange. Short read alignments are in red. AMR genes mapping to *Pseudomonas aeruginosa* or any *Pseudomonas* species are highlighted in blue and purple, respectively. *Sample from day 12 did not have enough sequencing reads but was plotted to maintain even scaling on the x-axis

To demonstrate the utility of the CZ ID mNGS and AMR modules for studying the longitudinal dynamics of infection, we analyzed serially collected lower respiratory RNA-seq data from a critically ill patient with respiratory syncytial virus (RSV) infection who subsequently developed ventilator-associated pneumonia (VAP) due to *Pseudomonas aeruginosa* [38, 39]. Analysis of microbial mNGS data using the CZ ID pipeline highlighted the temporal dynamics of RSV abundance, which decreased over time. Following viral clearance, we noted an increase in reads mapping to *P. aeruginosa* on day 9, correlating with a subsequent clinical diagnosis of VAP and bacterial culture positivity (Fig. 7A) [38, 39]. Analysis using the CZ ID AMR module demonstrated that *P. aeruginosa*-associated AMR genes were also detected, and their prevalence tracked with the relative abundance of the nosocomial bacterial pathogen (Fig. 7B).

### Application 5: AMR gene detection from environmental surveillance samples

**Fig. 8:**
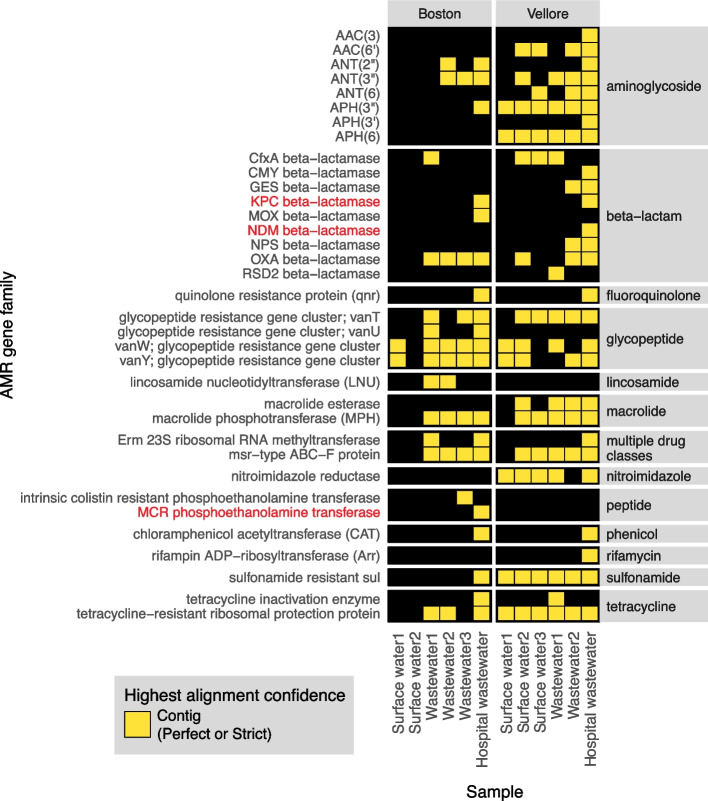
AMR surveillance from environmental water samples. AMR gene families identified from global surveillance of surface or wastewater samples from Boston, USA and Vellore, India. AMR genes found by contigs that passed Perfect or Strict cutoff are included in heatmap. Gene families of high public health concern are highlighted in red

Lastly, to highlight the application of the CZ ID AMR module for environmental surveillance of AMR pathogens, we analyzed publicly available Illumina mNGS data from a wastewater surveillance study comparing Boston, USA to Vellore, India [40]. In this study, municipal wastewater, hospital wastewater, and surface water samples were collected from each city and underwent DNA mNGS. From AMR gene alignments at the contig level, we observed a total 22 AMR gene families in Boston samples versus 30 from Vellore (Fig. 8). Several AMR genes of high public health concern such as those encoding the KPC and NDM plasmid-transmissible carbapenemases were only present in hospital effluent, reflecting the fact that hospitals frequently serve as reservoirs of AMR pathogens [43].

## Discussion

Metagenomics has emerged as a powerful tool for studying and tracking AMR pathogens in a range of research and public health contexts. Both surveillance and research applications of mNGS benefit from simultaneous assessment of AMR genes and their associated microbes, yet traditionally separate bioinformatics workflows and resource-intense computational infrastructure have been required for each. Here, we address these challenges with the CZ ID AMR module, a fast and openly accessible platform for combined analysis of AMR genes and microbial genomes that couples the expansive database and advanced RGI software of CARD with the unbiased microbial detection capacity of CZ ID. We demonstrate the AMR module’s diverse applications from infectious disease research to environmental monitoring through a series of case studies leveraging four observational patient cohorts and a wastewater surveillance study.

The CZ ID AMR module is designed to enable rapid and accessible data processing without a need for coding expertise, and return a comprehensive set of metrics to aid in data interpretation. Researchers can then apply stringency threshold filters to maximize sensitivity or specificity depending on the use case. For instance, when seeking to detect established AMR genes from data types with high coverage of microbial genomes (e.g., WGS data of cultured isolates), “Perfect” or “Strict” stringency thresholds maximize the accuracy of assignments. In contrast, from mNGS data with sparse microbial genome coverage (e.g., from blood or wastewater), using “Nudged” to increase sensitivity of mapping reads at the expense of specificity may be the only way to detect biologically important AMR genes. The “Nudged” threshold also enables more alignment permissiveness to sequence variations, which can be helpful for detecting novel alleles. The CZ ID AMR module provides various metrics to support optimization of cutoffs based on specific sample types and applications by the users.

Depending on the number of reads, breadth of coverage, and whether reads originate from conserved versus variable gene regions, the confidence of AMR gene assignment can vary. Generally, the confidence of contig-based AMR gene assignments is greater than read-based AMR gene matches due to the increased length of assembled fragments. When it comes to AMR alleles with high sequence similarity, such as those from within the same gene family, the AMR module can only distinguish between them if sufficient gene coverage is achieved. If genes within the same family are identified at both the individual read and contig level, preferentially evaluating the contig annotation will maximize allele assignment specificity.

With respect to limitations of the CZ ID AMR module, it is important to consider that the confidence of AMR and pathogen-of-origin calls depends on the completeness and accuracy of the CARD database. As our understanding of AMR gene biology increases over time, annotations may change in the CARD reference database that underpins the CZ ID AMR gene module. This was evident, for instance, in the *Klebsiella* transfusion-related sepsis case (Application 1, Fig. 3B), where *mdfA* was annotated as conferring resistance to tetracycline antibiotics based on CARD version 3.2.6, used for our analysis. This may be updated as a multiple drug resistance gene [44] in the future CARD releases. To mitigate database limitations and ensure traceability of results over time, CZ ID highlights the specific versions of the underlying databases used for each analysis.

Another limitation to consider is the downsampling of reads that occurs after host reads removal, which is done to accelerate pipeline processing speed. After removing low quality reads, host reads, and duplicate reads, the remaining reads are subsampled to 1 million for single-end reads or 2 million for paired-end reads. While downsampling has minimal impact on samples from which reads derive primarily from host (e.g., respiratory samples), it may reduce the sensitivity for detecting low abundance taxa or AMR genes from samples composed mostly of bacteria (e.g., stool samples). The compositional nature of samples thus should be considered in cases where AMR gene detection sensitivity is a priority.

The pathogen-of-origin prediction feature, designed to identify the source species of detected AMR genes, is still under development and thus should be interpreted with discretion. Pathogen predictions are based on matching AMR sequences in each sample to CARD Resistomes, Variants, & Prevalence database, and are best interpreted in the context of the microbes found to exist in the sample from the CZ ID mNGS module output. Connecting AMR genes to their exact originating microbes using short read sequencing data remains both a challenge and important area of active research.

Finally, sustainability in terms of maintenance and funding is a common limitation of most web-based bioinformatics tools and pipelines. As a commitment to preserving the functionality and modules that CZ ID currently provides, all underlying code for the platform is open-source. Additional future directions for the AMR module could include accommodating long read sequencing data, enabling the comparison of sequence variation between alleles of a given AMR gene, and improving capabilities for predicting genomic location of AMR genes.

## Conclusions

In sum, we describe the novel AMR analysis module within the CZ ID bioinformatics web platform designed to facilitate integrated analyses of AMR genes and microbes. This open-access, cloud-based pipeline permits studying AMR genes and microbes together across a broad range of applications, ranging from infectious diseases to environmental surveillance. By overcoming the significant computing infrastructure and technical expertise typically required for next-generation sequencing data processing, this tool aims to democratize the analysis of microbial genomes and metagenomes across humans, animals, and the environment.

## Supplementary Information

Additional file 1: Supplementary methods for sample and data processing, and Fig. S1 showing a detailed diagram of the CZ ID AMR and mNGS workflows

## Data Availability

All raw microbial sequencing data supporting the conclusions of this article are available via NCBI’s Sequence Read Archive under BioProjects PRJNA544865 (https://www.ncbi.nlm.nih.gov/bioproject/?term=PRJNA544865) [35], PRJNA450137 (https://www.ncbi.nlm.nih.gov/bioproject/?term=PRJNA450137) [36], PRJNA672704 (https://www.ncbi.nlm.nih.gov/bioproject/?term=PRJNA672704) [40] and PRJNA1086943 (https://www.ncbi.nlm.nih.gov/bioproject/?term=PRJNA1086943) (this paper). Results from CZ ID are in a public project called “AMR example applications” in https://czid.org which can be found through searching. CZ ID workflow code can be found in https://github.com/chanzuckerberg/czid-workflows/. Additional code for data filtering and plotting can be found in https://github.com/chanzuckerberg/czid-amr-manuscript-2024.

## Abbreviations

AMR: Antimicrobial resistance
ARO: Antibiotic Resistance Ontology
AWS: Amazon Web Services
CARD: Comprehensive Antibiotic Resistance Database
mNGS: Metagenomic next-generation sequencing
MRSA: Methicillin-resistant *Staphylococcus aureus*
MSSA: Methicillin-susceptible *Staphylococcus aureus*
NICU: Neonatal intensive care unit
RGI: Resistance Gene Identifier
RSV: Respiratory syncytial virus
SNP: Single nucleotide polymorphisms
WDL: Workflow Description Language
WGS: Whole-genome sequencing
VAP: Ventilator-associated pneumonia
