# Supplementary material for: Simultaneous detection of pathogens and antimicrobial resistance genes with the open source, cloud-based, CZ ID platform
Source: Genome Med. 2025 May 6;17:46. doi: 10.1186/s13073-025-01480-2 (PMC12057172; doi:10.1186/s13073-025-01480-2)
Supplement: Supplementary file 1 — Additional file 1: Supplementary methods for sample and data processing, and Fig. S1 showing a detailed diagram of the CZ ID AMR and mNGS workflows [file 13073_2025_1480_MOESM1_ESM.docx]

### **Supplementary Materials**

**Methods**

**Nucleic acid extraction and Illumina sequencing**

For the skin swab samples and cultured isolates described in Application 2, DNA was extracted using the Zymo Pathogen Magbead kit (Zymo Research) according to manufacturer’s instructions. Sequencing libraries were then prepared from 20ng of input DNA using the NEBNext Ultra-II DNA kit (New England Biolabs) following manufacturer’s instructions [1]. For the tracheal aspirate samples described in Application 4, RNA was extracted using the Qiagen Allprep kit (Qiagen) following manufacturer’s instructions. Sequencing libraries were prepared using the NEBNext Ultra-II RNA kit (New England Biolabs) according to a previously described protocol [2]. Paired end 150 base pair illumina sequencing was performed on all samples using Illumina NextSeq 550 or NovaSeq 6000.

**AMR gene identification**

We downloaded the tabular results from the CZ ID AMR module (the project can be accessed at <https://czid.org/home?project_id=5929> after logging in) and applied quality filters to ensure robust AMR gene identification. Specifically, for mNGS data, we required all AMR genes (from contig and read approaches) to have coverage breadth > 10% and for read mappings we additionally required > 5 reads mapping to the AMR gene. For single-isolate WGS data, we required all AMR genes (from contig and read approaches), to have coverage breadth > 50% and additionally required > 5 reads mapping to the AMR gene for read results. Across all analyses, Nudged results were treated the same way as contig results. For studies with corresponding water controls, we applied the above filters to the water controls, and then removed AMR genes or gene families (depending on what was plotted) also found in water controls from experimental samples.

**AMR gene heatmaps**

All plots were generated in R using Tidyverse [3], patchwork [4] and ComplexHeatmap [5]. While making the plots, we did an additional filtering to focus the analysis within the context of the use-case and limit the size of the plots for the paper. In particular, we included only CARD’s protein homolog and protein variation models (see <https://github.com/arpcard/rgi>), and included only medically relevant antibiotics drug classes by removing disinfecting agents and antiseptics, antibacterial free fatty acids, and aminocoumarin, diaminopyrimidine, elfamycin, fusidane, phosphonic acid, nucleoside, and pleuromutilin antibiotics. In Fig. 5B and Fig. 8, we also excluded efflux pumps to reduce plot size.

Then, we applied a series of heuristics to make this structured data amenable to heatmap visualization. Given the nature of a heatmap visualization, each AMR annotation in each sample can have only one representing tile, so we plotted the result with the highest confidence: we considered AMR genes identified through the contig approach with Perfect or Strict cutoffs as higher confidence than those with the Nudged cutoff, which were then of higher confidence than AMR genes found by reads alone. Finally, given the challenges for gene attribution presented by homology between genes in the same gene family, we developed a systematic approach for collapsing the visualization to a single AMR candidate per gene family per sample. For all figures except for Fig. 6, if in the same sample one AMR gene was found by the read approach and a different AMR gene from the same gene family was found by the contig approach, the first AMR gene was omitted and only the second AMR gene was plotted. The rationale for this prioritization stems from the fact that sometimes short reads alone cannot sufficiently distinguish between highly similar alleles or genes from the same gene family. Contigs, which typically provide greater sequence length are often of higher confidence. This approach should be considered on a per gene or per gene family basis, due to variability in the extent of sequence similarity within genes and gene families, and also be modified for specific use cases. For example, in Fig. 6B, even though *mecR1* and *mecA* are from the same gene family, they do not have highly similar sequences and we did not apply this step.

**Species identification**

For results from the CZ ID mNGS module, filters were again applied to ensure high-quality results. Specifically, for Fig. 3 and Fig. 7, which each focused on a single species, the NT rpM calculated by the mNGS module was used with no extra filtering. For Fig. 5 and Fig. 6A, which focused on species composition, the species detected by the mNGS module were filtered with: NT rpM > 10 and NR rpM > 10 to implement a minimal abundance requirement for taxonomic identification, NT alignment length > 50 to ensure alignment specificity and NT Z-score > 2 using a background model calculated with the corresponding study-specific water samples to ensure significance of taxa above levels of possible background contamination. Finally, for Fig. 6B, which had low read coverage, abundance filters were omitted and only the significance filter of NT Z-score > 2 was applied, using a background model calculated with the corresponding water samples.

**SNP distance analysis**

Host-filtered reads were downloaded from the CZ ID mNGS module. SNP distance were calculated with SKA2 0.3.2 [6] using ska build --min-count 4 --threads 4 --min-qual 20 -k 31 --qual-filter strict and ska distance --filter-ambiguous. The heatmap plot was generated with ComplexHeatmap [5] using default distance (“Euclidean”) and hierarchical clustering method (“complete").

**Software versions**

The following software versions were used for this manuscript: CZ ID mNGS workflow version 8.2.5, CZ ID AMR workflow version 1.4.2 based on CARD RGI version 6.0.3, CARD database versions 3.2.6 and the CARD Resistomes, Variants & Prevalence database: 4.0.0 [7]. SKA2 version 0.3.2 [6].


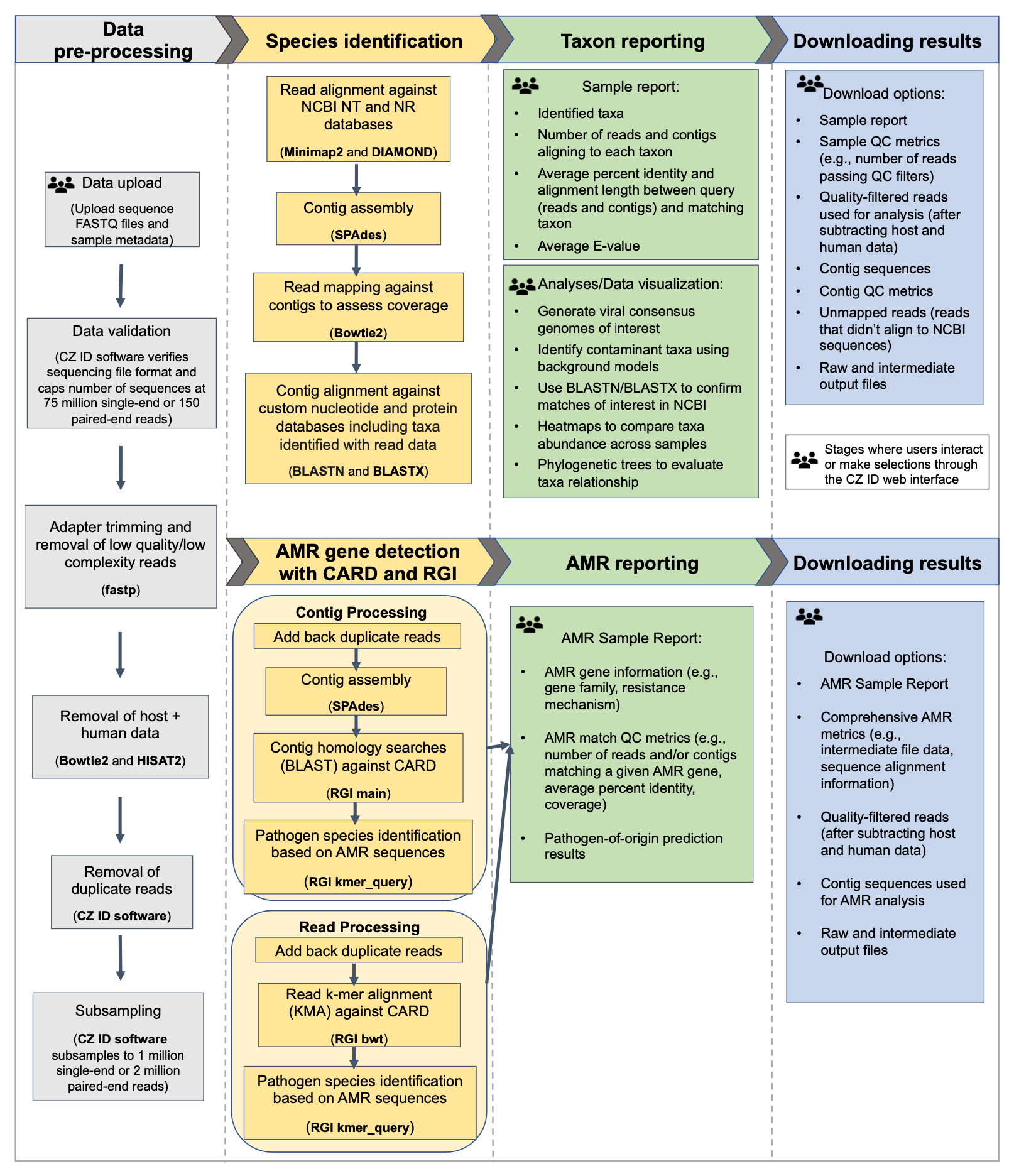


**Fig. S1. Detailed flow diagram highlighting the integrated AMR and mNGS modules within the CZ ID workflow.**

### **References:**

1. Crawford E, Kamm J, Miller S, Li LM, Caldera S, Lyden A, et al. Investigating Transfusion-related Sepsis Using Culture-Independent Metagenomic Sequencing. Clin Infect Dis. 2020;71:1179–85.

2. Tsitsiklis A, Osborne CM, Kamm J, Williamson K, Kalantar K, Dudas G, et al. Lower respiratory tract infections in children requiring mechanical ventilation: a multicentre prospective surveillance study incorporating airway metagenomics. Lancet Microbe. 2022;3:e284–93.

3. Wickham H, Averick M, Bryan J, Chang W, McGowan LD, François R, et al. Welcome to the Tidyverse. Journal of Open Source Software. 2019;4:1686.

4. Thomas Lin Pedersen. patchwork: The Composer of Plots. R package version 1209000, https://github.com/thomasp85/patchwork [Internet]. 2024; Available from: https://patchwork.data-imaginist.com

5. Gu Z, Eils R, Schlesner M. Complex heatmaps reveal patterns and correlations in multidimensional genomic data. Bioinformatics. 2016;32:2847–9.

6. Derelle R, Wachsmann J von, Mäklin T, Hellewell J, Russell T, Lalvani A, et al. Seamless, rapid, and accurate analyses of outbreak genomic data using split k-mer analysis. Genome Res. 2024;34:1661–73.

7. Alcock BP, Huynh W, Chalil R, Smith KW, Raphenya AR, Wlodarski MA, et al. CARD 2023: expanded curation, support for machine learning, and resistome prediction at the Comprehensive Antibiotic Resistance Database. Nucleic Acids Res. 2023;51:D690–9.
